# Cytotoxic antibodies – valuable prognostic factor for long term kidney allograft survival

**Published:** 2010-11-25

**Authors:** AM Moise, D Nedelcu, A Toader, M Sora, A Tica, DE Ferastraoaru, I Constantinescu

**Affiliations:** * ‘Carol Davila’ Medical University, Immunology of Transplantation, Fundeni–Centre for Immunogenetics and Virology, Bucharest Romania; ** Biologist – Immunology of Transplantation, Fundeni–Centre for Immunogenetics and Virology, BucharestRomania; *** Clinical immunology and allergology, ‘Nicolae Malaxa’ Clinical Hospital, BucharestRomania; **** Virology and Immunology, ‘Carol Davila’ Medical University, Fundeni–Centre for Immunogenetics and Virology, Bucharest Romania

**Keywords:** renal transplant, anti–HLA sensitisation, cytokines

## Abstract

Background: Since the first attempts of kidney transplant, the inflammation mediated by T lymphocytes was considered one of the most important processes implicated in graft rejection but, multiple acute and chronic graft rejects revealed that the inflammation process is not singular and humoral mechanisms may play a role in the development of chronic vascular rejection.

Material and methods: We evaluated 500 Romanian patients registered on the kidney transplant waiting list. We performed anti–HLA class Ⅰ and class Ⅱ antibodies screening and identification. Laboratory tests were performed at Centre for Immunogenetics and Virology,  Clinical Institute Fundeni, Bucharest, Romania. The methods used are represented by ELISA (GTI Diagnosis, USA) and Luminex (Tepnel, USA)

Results: pretransplant evaluation of the subjects illustrates that 145 patients (29%) have been sensitized and 355 patients (71%) have not been sensitized. The most frequent types of anti–HLA antibodies were: A2 (13%),  B42 (10%),  DR7 and DR11 (13%). Posttransplant, the most cases with de novo antibodies were observed in the first 6 months post transplantation. High serum levels of Il–2Receptor, TNF–alpha and neopterin in post transplant sensitized patients were observed following de novo cytotoxic antibodies occurrence.

Conclusion: post renal transplantation, patients present high risk in developing de novo cytotoxic antibodies, especially those who had HLA mismatch with the donor. These antibodies are predictors for acute graft rejection and for graft failure.

## Introduction

### Generalities

The first successful kidney transplantation has been performed in Boston in 1954 between genetically identical twins. In Romania, the first renal transplant was carried out by Professor Dr. Eugen Proca in 1980 at Fundeni Clinical Institute. In 1997 has been made the first cadaveric renal transplant following multiple organs harvesting. In 1998, the first renal transplant in a diabetic patient was achieved. In the same year the first kidney transplant in an adult with chronic renal insufficiency due to cancer was performed. The performances continued and in 1999, the first kidney transplant in a child from a deceased person took place. In 2005 the first renal transplant in an anephric child due to bilateral Wilms tumor was performed.

Since the first attempts of kidney transplant, the inflammation mediated by T lymphocytes was considered one of the most important processes implicated in graft rejection. Therefore, anti rejection therapies were directed against T lymphocytes. By using these therapies has decreased the rate of acute rejection and has significantly increased post transplant survival at one year. Nevertheless, the multiple acute and chronic graft rejects revealed that the inflammation process is not singular. Immunohistochemical methods to visualize the complement reveal, at the graft, antibodies depositions and consecutive complement activation [1].

HLA–A, HLA–B, HLA–DR compatibility does not guarantee kidney transplant free of rejection. At this moment, anti–HLA antibodies detection and identification represents one of the most important points in transplant research. 

### The HLA system

The human leukocyte antigen system (HLA) is the name of the major histocompatibility complex (MHC) in humans. The superlocus contains a large number of genes related to immune system function in humans. This group of genes resides on chromosome 6 and encodes cell–surface antigen–presenting proteins and many other proteins. MHC molecules are divided into 2 main classes: HLA class I antigens (HLA–A, HLA–B, HLA–C) – presented on the surface of all nucleated cells and platelets and HLA class II antigens (HLA–DR, HLA–DQ, HLA–DP, HLA–DM, HLA–DO) –  expressed on professional antigen–presenting cells, but also on the surface of endothelial vascular cells and renal tubular epithelial cells [2]. Due to high polymorphism degree of HLA system (more than 1600 alleles), the individuals do not present identical sets of HLA molecules. HLA polymorphism confers immunological identity to each person and thus, the immune system can differentiates between ‘self’ and ‘non self’. The involvement of HLA system in acceptance or rejection of the transplanted organ, represents a collateral consequence of its main function (in terms of immunological, a transplanted organ represents an immense pathological extracellular product)[3].

Before the era of new immunosuppressive drugs, transplantation in patients who had HLA configuration as close to the donor is accompanied by a better graft survival than less compatible recipient–donor pairs.However, the allocation of kidneys based on HLA match (especially for unrelated donors) may constitute a disadvantage for patients with a rare, unusual HLA phenotype. Due to high degree of HLA polymorphism raising chances of immunisation because HLA molecules recognized as ‘no self’ represent targets for the immune system. Cytotoxic antibodies (anti–HLA) does not occur naturally but after the blood/plasma transfusion, in pregnancy or after a previous transplant. Usually, the transfused women and with multiple pregnancies have high degree of sensitization by developing antibodies against multiple HLA antigens types [9]. When these antibodies are directed against HLA system of transplanted organ, their targets are represented by the graft endothelial cells. Next step is activation of complement, coagulation cascade and other inflammation factors, having as the final result severe injury of endothelial cells and dysfunction of the transplanted organ. This mechanism mediated by anti–HLA antibodies is called humoral rejection (hyperacute rejection). Thus, detection and ability to correctly identify these antibodies in sera of patients highly sensitized before transplantation is an important stage in detemining immunological risk for recipient and for incompatible donor exclusion [4]. In the past, cytotoxic antibodies identification has been impaired by: (1) inability to distinguish between IgM and IgG antibodies; (2) correct identification of anti–HLA class Ⅱ antibodies, especially in the  presence of anti–HLA class Ⅰ antibodies; (3) inability to detect the antibodies hidden by linkage disequilibrium. For example, in case of lymphocytotoxic tests, if all cells from a panel have  HLA–B8 and HLA–A1 phenotypes, due to linkage disequilibrium, it is difficult to say that the patient has anti–HLA–B8 or anti–HLA–A1 antibodies or both antibodies types. 

These problems were overcome by developing screening and identification methods based on solid phase multiplex platforms (ELISA, flow cytometry, luminex) that can detect binding of serum antibodies to specific antigens independently of  complement activation. Also, with these new technologies, using additional anti–IgM/IgG antibodies,  is possible to distinct between IgM and IgG anti–HLA antibodies. Additionally, it can discriminate between HLA class Ⅰ and class Ⅱ antibodies and more,  single antigen methods allow identification of an unique HLA specificity

In 1969, Patel and Terasaki demonstrated the clinical importance of anti–HLA antibodies. In present is very well known that the recipients who present preformed cytotoxic antibodies have a high rate of graft rejection [5].  It is also true that the individual importance of anti–HLA class Ⅰ and class Ⅱ antibodies to graft rejection is incompletely understood. Some studies show that only the presence of both types of antibodies is accompanied by a decrease in graft survival, whereas isolated reactivity have not clinical consequences [10, 11]. On the other hand, several recently reported cases have shown that anti–DP antibodies are a potential risk factor for graft dysfunction and failure. HLA–DP mismatch between donor and recipient does not influence graft function at the first kidney transplant but has an negative impact in case of a retransplant [12, 13, 14].

Binding of specific antibodies to antigens on the vascular endothelium followed by complement activation represents the main mechanism underlying acute and hyperacute rejection. Complement system is activated by classical pathway having as final result the membrane attack complex  MAC, which lyses endothelial cells followed by graft rejection [6].

The aim of our study was the systematic pursuit of class Ⅰ and/or class Ⅱ cytotoxic antibodies pretransplant, their de novo appearance posttransplant (donor or nondonor–specific) and integrating these results with serum levels of cytokines (TNF–alpha, IL–2 Receptor, neopterin) and immunosuppressants in order to assess the patient's immunological status and subclinical or acute rejection episodes. 

## Material and methods

We evaluated a total of 500 Romanian patients registered on the waiting list for renal transplant who subsequently received kidney transplantation. We performed anti–HLA class Ⅰ and class Ⅱ antibodies screening in all subjects and in case they were present their identification was made. Laboratory tests were performed at Centre for Immunogenetics and Virology, Clinical Institute Fundeni, Bucharest, Romania. The methods used are represented by ELISA (GTI Diagnosis, USA) and Luminex (LifeScreen– Tepnel, USA).

ELISA is a qualitative solid phase enzyme linked immunosorbent assay in that patient serum is added to microwells coated with affinity purified HLA class Ⅰ or class Ⅱ glycoproteins allowing antibodies, if present, to bind. Unbound antibodies are then washed away. An alkaline phosphatase labeled anti–human globulin reagent (anti–IgG) is added to the wells and incubated. The unbound anti–IgG is washed away and the substrate PNPP (p–nitrophenyl phosphate) is added. After a 30 minutes incubation period, the reaction is stopped by a sodium hydroxide solution. The optical density of the color that develops in each microwell is measured in a spectrophotometer and compared to a cutoff value.

LifeScreen is composed of unique Luminex Beads to which affinity purified HLA class Ⅰ and class Ⅱ HLA glycoproteins are conjugated. An aliquot of the beads is allowed to incubate with a small volume of patient serum. The sensitized beads are then washed to remove unbound antibodies. An anti–human IgG antibody conjugated to phycoerythrin is then added. After another incubation, the test sample is diluted and analyzed on the Luminex instrument based on  two laser lines. One laser identifies each bead and the other captures the fluorescent signal emitted by phycoerythrin. The signal intensity from each bead is compared to the signal intensity of a negative control bead included in the bead preparation to determine if the bead is positive or negative for bound alloantibodies.

In  addition, postrenal transplant, in patients who had de novo cytotoxic antibodies we measured serum levels of TNF–alpha, IL–2 receptor and neopterin by enhanced chemiluminescence method using Immulite device. The Immulite system assay utilizes specific antiboies or antigens coated plastic beads as the solid phase, alkaline phosphatase labeled reagent and chemiluminescent enzyme substrate. After incubating the sample with the alkaline phosphatase  reagent, the liquid reaction mixture in the Test Unit is rapidly separated from the beads when the beads are washed and the Test Unit is spin at a high speed on its vertical axis. The entire fluid contents is transferred to a coaxial waste chamber in the Test Unit. The beads are left with no residual, unbound label. The bound label is then quantitated with a dioxetane substrat, which produces light. Light emission is detected by a photomultiplier tube and printed report for each sample are generated by system's computer. 

## Results

**Figure 1:**
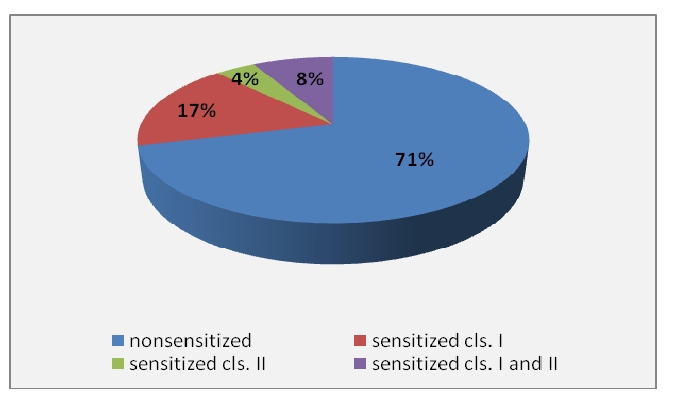
Anti– HLA class Ⅰ and class Ⅱ antibodies distribution in study group

Pretransplant evaluation of the total of 500 subjects included in our study illustrates that 355 patients (71%) have not been sensitized, 85 patients (17%) presented anti–HLA class Ⅰ antibodies, 19 patients (4%) presented anti HLA class Ⅱ antibodies and in 41 subjects (8%)  we discovered both types of antibodies. (Figure 1)

In sensitized patients, next stage was the identification of cytotoxic antibodies specificity. Generally, the antibody specificity is  to a certain epitope (antigenic determinant) represented by a short proteic sequence (6–10 amino acids) of an antigen structure, sequence that is recognized by the T or B–cell receptor. Since HLA molecule are a very complex structures, with more than 300 amino acids, it is not surprising they contain multiple alloepitopes that are capable of inducing a cellular or humoral immune response. Epitopes that originally were thought to occur only on a single gene product such as HLA–A2 were reffered to as private epitopes. Other anti–HLA antibodies that reacted with more than one gene product were throught to detect a shared or crossreactive epitope, termed public epitopes. Many of the public epitopes are  widely distributed among HLA molecules. Antibodies to public epitopes have been used to categorize HLA gene products into major cross–reactive groups (CREG's).  

**Figure 2:**
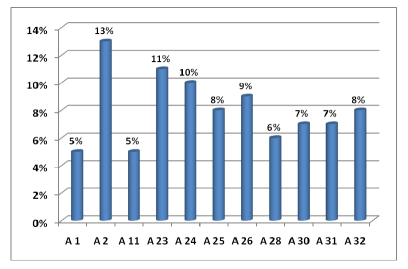
Anti–HLA–A cytotoxic antibodies distribution

**Figure 3:**
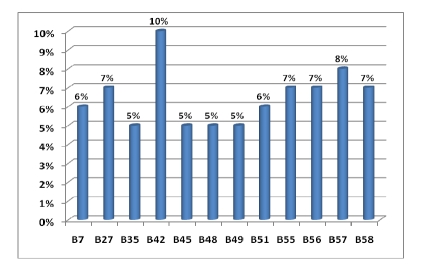
Anti–HLA–B cytotoxic antibodies distribution

**Figure 4:**
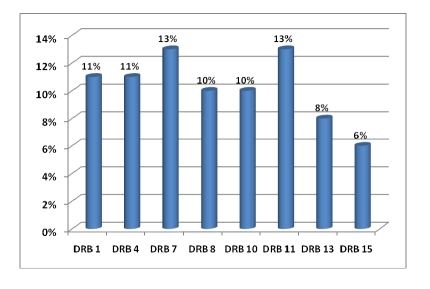
Anti–HLA–DR cytotoxic antibodies distribution

In Figure 2, Figure 3 and Figure 4 are the percentage of anti–HLA specificities found in patients in our study group.

In our study the most frequently anti–HLA specificities  were:

- A2 (13%) followed by A23 and A24 (11 si 10%) then A25, A26, A32 (about 9%);
- B42 (10%), B57 (8%), B27, B55, B56, B58 each with 7 percent;
- DR7 and DR11 (13%), DR1 and DR4 (11%), DR8 and DR10 (10%).

This distribution is not surprising since the corresponding antigens of these antibodies are among the most common in the Romanian population and therefore the chance that a patient's immune system to be sensitized towards these antigens is very high.

At 1 year posttransplant, among the initially nonsensitized patients, 303 who had more than 50% compatibility af HLA–A, B, DRB1 with donor (ie, had at least three of the six identical alleles) developed de novo cytotoxic antibodies in a proportion of 13%  (39 patients) while the remainder (52 patients) in which donor–recipient compatibility was less than 50%,  de novo antibodies occurred at a rate of 27% (14 recipients).

In previously immunized patients, the percentage who developed de novo posttransplant cytotoxic antibodies was slightly higher, 32% (46 patients), evidenced by PRA (Panel Reactive Antibody) increased.

Most cases of de novo antibodies appearance were observed in the first 6 months post transplantation. 86% of transplant recipients developed at least one episode of acute reject controlled by immunosuppressive therapy. We observed that patients under immunosuppressive treatment with Prograf (tacrolimus) and MMF (mycophenolate mofetil) presented decreased rates of cytotoxic antibodies production compared with patients under treatment with CsA (cyclosporine A) and MMF (mycophenolate mofetil). In addition, subjects under immunosuppressive treatment with Rapamune (Sirolimus) and MMF (mycophenolate mofetil) presented higher rates of cytotoxic antibodies production.

Colvin et al. [	15] revealed that serial monitoring of serum levels of Il–2 Receptor (Il–2R) could have diagnostic value for detection of posttransplant kidney rejection. Beutler and Cerami [16] detected high levels of serum TNF–alpha during acute rejection and infections periods suggesting that TNF–alpha could represent a relevant mediator of transplant reject. Sometimes, elevated TNF–alpha were found before creatinine levels increase and then TNF–alpha fell rapidly, in 2–3 days after intensive immuosuppressive therapy. There are other studies which reveal that serum levels of neopterin are persistently high after renal transplantation in uremic patients with acute tubular necrosis. In addition, daily measurement of this parameter could be useful for biochemical detection of immunological complications. [17]

**Figure 5:**
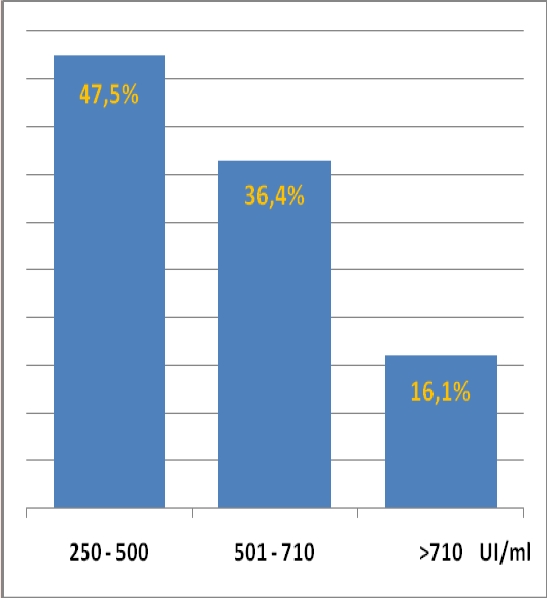
IL–2 R serum levels in posttransplant sensitized patients, (normal range = 223 – 710UI/ml)

**Figure 6:**
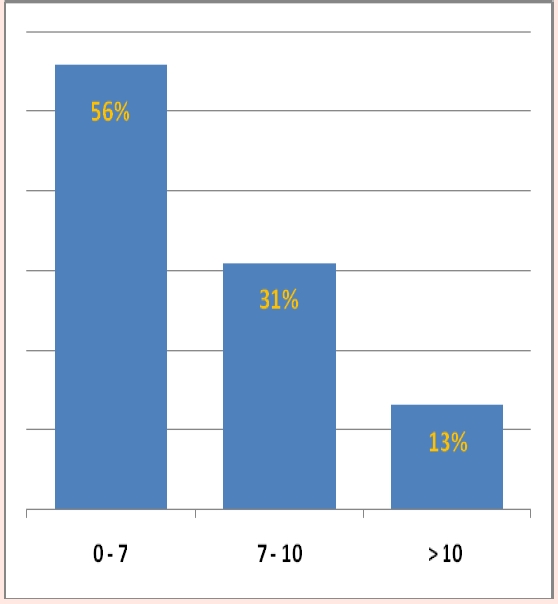
TNF–alpha serum levels in posttransplant sensitized patients, (normal range = 0 – 8,1 pg/ml)

**Figure 7:**
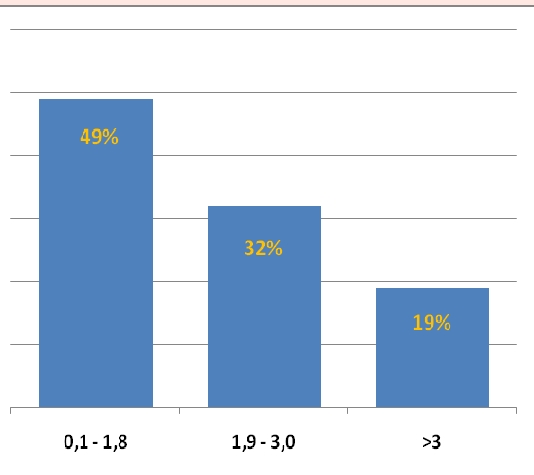
Neopterin serum levels in posttransplant sensitized patients, (normal range = 0,3 – 3,0 ng/ml)

In our group of posttransplant sensitized patients (n=99), serum levels of IL–2R, TNF–alpha and neopterin were in the value ranges represented in the graphs in Figure 5, Figure 6 and Figure 7 and high levels were evidenced after a variable period of time (2–15 days) following de novo HLA antibodies occurrence.

## Discussions

In recent years, there is an increased interest in the study of cytotoxic antibodies involved in graft rejection in kidney transplantation. In literature, a rate of about 20–25% of subjects undergoing a kidney transplant shows cytotoxic antibody increased titers. In our study, the percentage was 19.8%. We found that both, compatible and incompatible subjects, postkidney transplantation could develop de novo anti–HLA antibodies, predictable for acute graft rejection. The most common types of HLA antibodies were anti–HLA–A2, 23, 24, HLA–B42, 57, 27, HLA–DR7, 11, 1, 4.  Important to note is that recipients who developed de novo anti–HLA antibodies associated with severe rejection phenomena exhibited increased serum levels of TNF – α, IL–2R and neopterin. Immunosuppressive therapy based on tacrolimus with Mycophenolate mofetil combination is more effective in preventing HLA alloimunization post renal transplantation than combinations that include cyclosporine or sirolimus associated with Mycophenolate mofetil. In conclusion, to note that, postkidney transplant, recipients have an increased risk of developing de novo cytotoxic antibodies, especially those had an HLA mismatch with donor, and these antibodies may be predictive for acute rejection episodes and graft failure. A correct monitoring algorithm postrenal transplantation  includes: (1) best possible HLA match, (2) detection and identification of cytotoxic antibodies (3) determining cytokine gene polymorphism in conjunction with their serum levels (4) monitoring of immunosuppressive therapy (5 ) assessment of  serum viral load (CMV, EBV, BKV polyoma, parvovirus B19, HBV, HCV).
